# Hepatoprotective effects of gamma-aminobutyric acid-enriched fermented *Hovenia dulcis* extract on ethanol-induced liver injury in mice

**DOI:** 10.1186/s12906-020-2866-0

**Published:** 2020-03-06

**Authors:** Na-Hye Park, Seung-Jin Lee, Abraham Fikru Mechesso, Naila Boby, Quah Yixian, Woong-Kyu Yoon, Sam-Pin Lee, Jong-Suk Lee, Seung-Chun Park

**Affiliations:** 1 College of Veterinary Medicine, Kyungpook National University, 80, Daehak-ro, Buk-gu, 41566 Daegu, Republic of Korea; 2 Department of Food Science and Technology, Keimyung University, Daegu, 42601 Republic of Korea; 3 Biocenter, Gyeonggido Business and Science Accelerator (GBSA), Suwon, Gyeonggi-do 16229 Republic of Korea

**Keywords:** Ethanol, Fermentation, Hepatoprotective, *Hovenia dulcis*, γ-Aminobutyric acid, Lipogenesis

## Abstract

**Background:**

Various extracts of *Hovenia dulcis* have been commonly used in Asia for cases of alcohol-related disorders. Fermentation is reported to enhance the level and biological activities of various bio-constituents of plant extracts. Therefore, this study was undertaken to evaluate the effects of fermented *H. dulcis* extract (FHDE) on ethanol-induced liver injury in mice.

**Methods:**

FHDE was prepared using *Bacillus subtilis* and *Lactobacillus plantarum.* The effects of FHDE on ethanol-induced liver injury were evaluated in C57BL/6 N CrSlc mice. A mixed feed preparation containing the fermented extract with and without ethanol was given to mice for 29 days, according to its group. At the end of the experiment, blood and liver samples were collected from all mice in the group. Plasma biochemical analysis and histopathological investigation were performed to evaluate the impacts of treatment on the biomarkers of hepatic damage and inflammatory changes. Besides, the expression of genes that regulate the activities of enzymes associated with alcohol metabolism, antioxidant activity, and fatty acid oxidation was assessed using a quantitative real-time polymerase chain reaction. Moreover, the amino acid contents and the active ingredients of the extract were evaluated before and after fermentation.

**Results:**

Fermentation resulted in a marked increase and decrease in the amount of Gamma-Amino-n-butyric acid (GABA) and glutamic acid, respectively. FHDE enhanced the body weight gain of mice compared to ethanol. Besides, plasma levels of triglyceride, low-density lipoprotein, the activities of aspartate aminotransferase (AST) and alanine aminotransferase (ALT) were significantly (*P < 0.05*) reduced in the FHDE-treated groups relative to the ethanol-treated control. FHDE upregulated the expression of genes associated with enzymes involved in alcohol dehydrogenation (*Adh1* and *Aldh2*), antioxidant activity (*SOD* and *CAT*), and fatty acid oxidation (*PPAR-α* and *PGC-1α*). However, the expressions of Cytochrome peroxidase *Cyp*_*2*_*E*_*1*_ and genes related to lipogenesis (*SREBP-1c*, *FAS*, *SCD-1*, and *ACC*) were significantly (*P < 0.05*) downregulated following treatment with the FHDE. Histopathological investigation demonstrated a slight degree of inflammatory cell infiltration and occasional fatty changes in the FHDE-treated groups.

**Conclusion:**

The GABA-enriched fermented *H. dulcis* extract prevented ethanol-induced hepatic damage by enhancing the antioxidant defense system, fatty acid oxidation, and reducing lipogenesis.

## Background

**Fig. 1:**
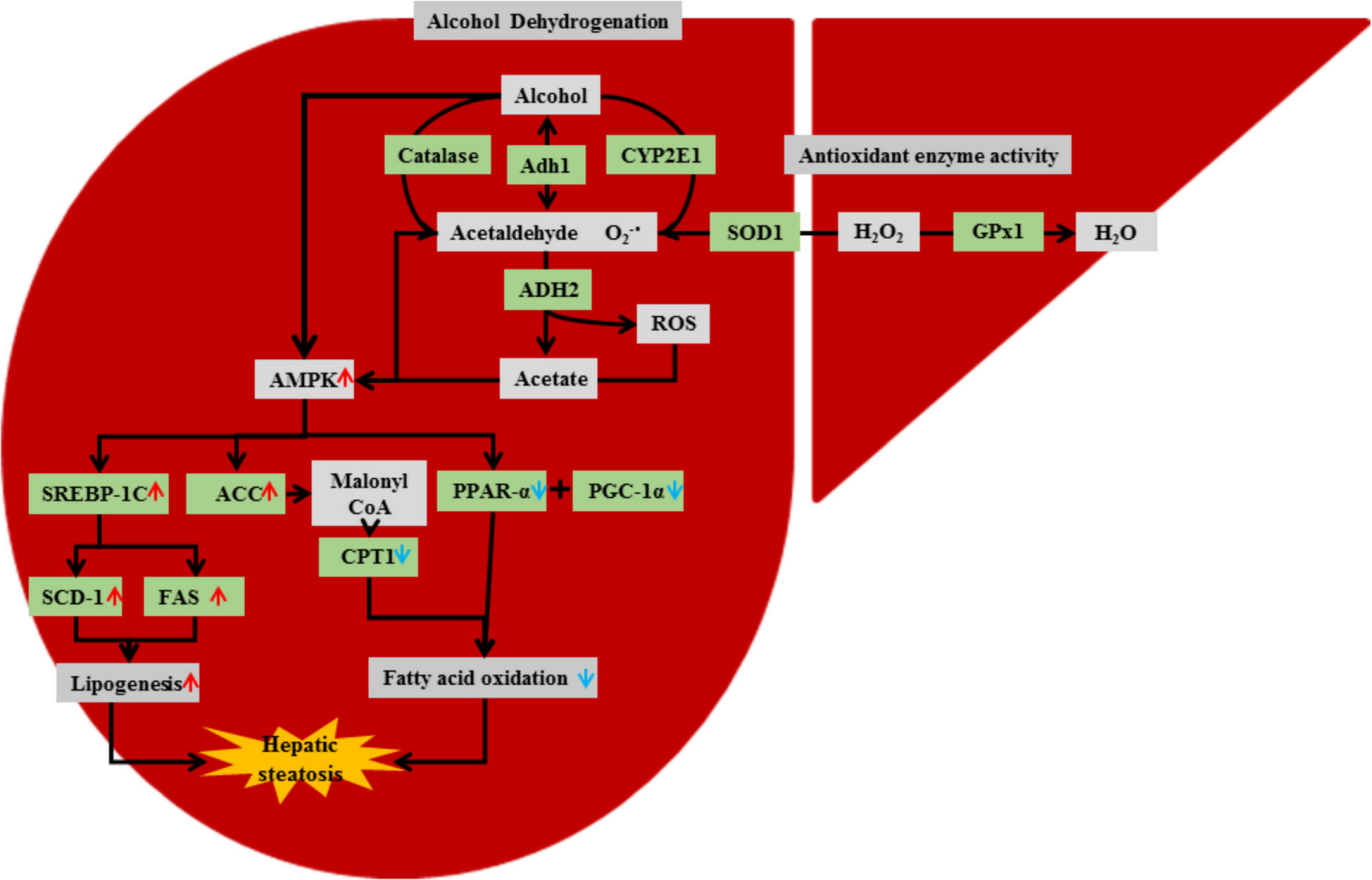
The figure demonstrates enzymes related to alcohol and lipid metabolism in the liver. Overconsumption of alcohol contributes to hepatic steatosis through activation and suppression of enzymes involved in lipogenesis (highlighted in red arrow) and fatty acid oxidation (highlighted in blue arrow), respectively

Excessive alcohol intake is one of the major causes of liver injury. It impairs the secretion of very low-density-lipoproteins from hepatocytes and predisposes to fatty liver [1]. Over-consumption of alcohol and subsequent metabolism results in the generation of reactive oxygen species, pro-inflammatory cytokines, and lipid peroxidation that collectively imposes steatosis, which can progress to cirrhosis and liver failure (Fig. 1) [2]. Therefore, agents that interfere with oxidative stress and lipogenesis are essential to avoid the development of alcohol-induced liver injury. Natural products with anti-inflammatory and anti-oxidant activities are becoming the target to develop therapeutic agents that can prevent the development of alcohol-induced liver injury [3].

Medicinal plants have been used widely in Asia for alcohol-related liver diseases. Nevertheless, none of them fully recovers the liver from its pathological conditions [4]. *Hovenia dulcis* is among those plants that are commonly used in Korean traditional medicine to relieve alcohol-related illnesses. The plant is proven to contain various metabolites, including, polyphenols, polysaccharides, vitamin C and flavonoids, which are related to immune-stimulatory, anti-lipidemic, anti-inflammatory, anti-adipogenic and anti-oxidant activities [5–7]. In China and Korea, various extracts of the plant are available in the market as tablets, powders, liquids or granules. These formulations have been used for the treatment of alcohol-related hepatic disorders and as dietary supplements [4, 8]. In vitro and in vivo studies have demonstrated the hepatoprotective [3, 9] and anti-adipogenic [5] activities of the crude extracts of *H. duclis.* Kim et al. [10] illustrated that *H. duclis* extracts significantly prevented alcohol-induced acute and chronic liver injury in mice. The extract also reduced the carbon tetrachloride (CCl_4_)-induced rise in the plasma levels of liver enzymes [10, 11].

Fermentation is reported to enhance the absorption, increase the level and biological activity of various bio-constituents of plant extracts [12, 13]. Despite the advantages of fermentation, only few studies have been conducted on the impacts of fermented extracts on alcohol-induced liver injury in mouse models. A study by Xiang et al. [14] demonstrated the impacts of fermented vinegar from *H. dulcis* on the antioxidant systems of mice exposed to alcohol. The fermented extract enhanced glutathione (GSH) content, total superoxide dismutase (T-SOD), catalase (CAT), and glutathione peroxidase (GSH-Px) activities. However, the study was conducted only on the vinegar from *H. dulcis* peduncles following a single-step fermentation. Besides, the effects of fermentation on the amino acid contents of the extract and its mechanisms of hepatoprotective effects were not exhaustively identified. In this study, the hepatoprotective effects of FHDE (probiotics based) were assessed in mice that feed on a diet containing ethanol. Further analysis was undertaken to determine the impacts of successive fermentation on the amino acid contents of *H. dulcis* extract.

## Methods

### Preparation of FHDE

The stem and leaves of *H. dulcis* were obtained from a certified company (Hambakjae Bio Farm Co., Ltd., Jeju Island, South Korea). The identity of *H. dulcis* was confirmed by a taxonomist (Dr. ZI-Eum Im) and voucher specimens (Voucher number KU-FST-010) were deposited at the Department of Food Science and Technology, Keimyung University, South Korea. The dried and crushed parts of the stem and leaves of *H. dulcis* were macerated with water (100 g in 1000 ml of water) for 8 h at 100 °C. A novel functional test product (FHDE) was prepared by co-fermenting 50 ml of the concentrated *H. dulcis* extract (HDE) with probiotics*.* Briefly, the extract was mixed with glucose (3%) and monosodium L-glutamic acid (5%), and autoclaved at 121 °C for 15 min. Then, *Bacillus subtilis* HA (KCCM 10775P) starter culture was inoculated and incubated at 42 °C for 3 days. Then, the product was mixed with skim milk (1%, v/v) and glucose (1.5%, v/v) solution. *Lactobacillus plantarum* EJ2014 (KCCM 11545P) was inoculated and incubated at 30 °C for 7 days for secondary fermentation. Finally, the fermented product was lyophilized via freeze dryer at − 70 °C for 3 days (Freeze Dryer, Ilshin BioBase Ltd., Ede, Netherlands; Pilot LP20).

### Liquid chromatography/mass spectrometry (LC/MS) and gas chromatography/mass spectrometry (GC/MS) analysis

The chemical compounds present in the HDE and FHDE were determined by LC/MS using an Accela UHPLC system (Thermo Fisher Scientific, CA, and USA) coupled with an LTQ-Orbitrap XL hybrid mass spectrometer (Thermo Electron, Bremen, Germany) via an ESI interface. Samples were separated using waters BEH C18 column (2.1 × 150 mm, 1.7 μm). The mobile phase consisted of distilled water (A) and acetonitrile (B) with 0.1% formic acid. The flow rate was set at 400 μL/min. The elution gradient was adjusted as follows: 5, 70, 100, and 100% acetonitrile for 1, 20, 24, and 27 min, respectively. Then, 1 μL of samples were injected and analysis was made in positive ion mode.

The GC-MS analysis was conducted using an Agilent gas chromatograph (Agilent Technologies, Santa Clara, CA, USA) and a 5975 GC-MS selective detector (Agilent Technologies). The column (30 m length × 0.25 mm internal diameter, and 0.25 m film thickness; Agilent J & B DB-5MS) temperature was maintained at 70 °C and 300 °C for 1 and 20 min, respectively. The analysis was conducted for a total of 6 h.

### Analysis of GABA content

The amino acid content of the HDE and FHDE was measured using an L-8800 amino acid autoanalyzer (Hitachi Ltd., Tokyo, Japan) following the manufacturer’s instructions. Briefly, 0.1 g of the fermented product was mixed with 5% trichloroacetic acid solution. The mixture was filtered through a 0.45-μm cellulose acetate filter paper. Then, the filtrate was diluted with 0.02 N hydrochloric acid. The analysis was conducted with a column packed with Hitachi custom Ion exchange resin (4.6 mm ID’ 60 mm L). The mobile phase was comprised of a buffer, PF (physiological fluid assay buffer) -1, 2, 3, 4, PF-RG (PF-regenerating solution, R-3, and C-1.The temperatures of the column and reactor were set to 50 °C and 135 °C, respectively. While the flow rate and detection wavelength were adjusted at 0.55 ml/min and 570 nm, respectively.

### In vivo experimental design

The experiment was carried out on male C57BL/6 N CrSlc mice (19–23 g in weight, 5 weeks old), obtained from Jung-Ang Animal Laboratory (Seoul, Korea). Mice were fed with a modified Lieber- DeCarli liquid diet (DYET# 710027, Dyets. Inc. USA). Mice were kept in the animal room with the temperature maintained at 25 ± 2 °C, humidity of 50–60%, and with a 12 h light/dark cycle. The experiment was conducted according to the international guidelines for the care and use of laboratory animals [15]. The total number of mice (*n* = 40) used in the present study was calculated by G*power program based on effect size (0.5), α-error probability (0.05), Power (1-β error probability) (0.6), and the number of groups (5). After 7-days of adaptation period, mice were arbitrarily divided into five groups, each consisting of eight mice. Treatment groups received 0.1% (FHDE1) and 0.3% (FHDE3) FHDE mixed with the diet containing 3% ethanol. Diet with and without ethanol (3%) was given to the negative (NC) and normal control (NRC) groups, respectively. HDE (0.3%: HDE3) mixed with the diet containing 3% ethanol was given to the positive control (PC) group. Mice were treated for a total of 29 days. Feed intake was monitored every other day. Whereas, body weight was measured once every 3 days. At the end of the experiment, mice were anesthetized by carbon dioxide inhalation following similar methods described by Mechesso et al. [16]. Blood was collected through cardiac puncture and immediately centrifuged at 4000x *g* for 5 min. Plasma was separated and stored at − 70 °C until use. In addition, half of the liver was preserved in formaldehyde for histopathological analysis and the remaining half was immediately processed for RNA extraction.

### Plasma biochemical analysis

The protective effects of FHDE from ethanol-induced liver damage was determined by measuring the activities of aspartate aminotransferase (AST) and alanine aminotransferase (ALT). Besides, the plasma levels of low-density lipoprotein (LDL), high-density lipoprotein (HDL), triglycerides (TG), and free fatty acid (FFA) were determined by using enzyme-linked immunosorbent assay kits (Sigma-Aldrich, St. Louis, MO, USA and Abcam, Cambridge, UK) following the manufacturer’s instructions.

### Extraction of total RNA and quantitative real-time PCR analysis

**Fig. 2:**
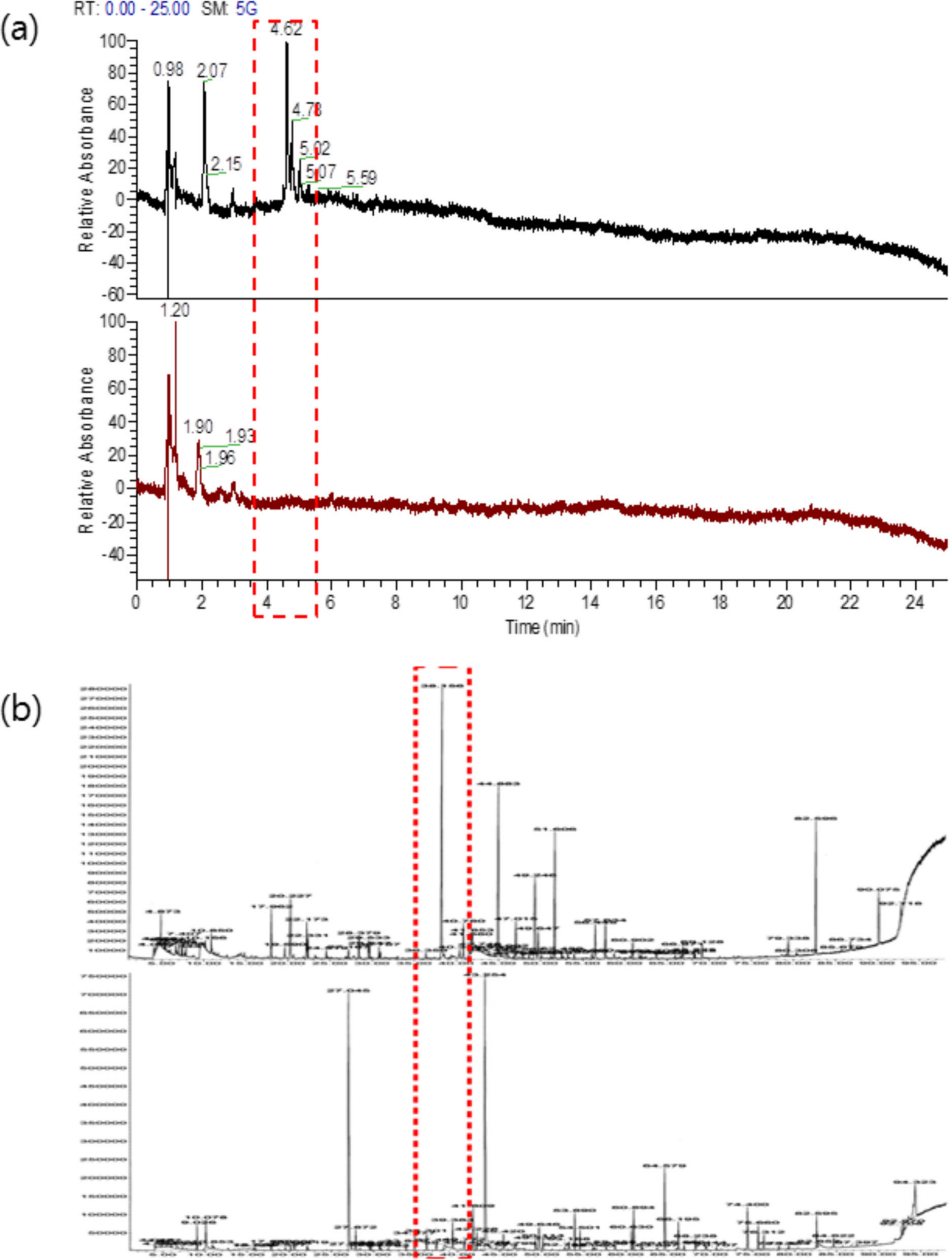
LC/MS (**a**) and GC/MS (**b**) chromatograms of HDE (upper) and FHDE (lower)

The effects FHDE on the expression of genes that are essential to regulate the activities of enzymes involved in alcohol and lipid metabolism were determined using quantitative real-time PCR (Fig. 2). For this purpose, a total of 50 mg liver tissue was homogenized (IKA T10 basic Homogenizer, Seoul, Korea) and total RNA was extracted using TRIzol (Ambion Life Technologies, Carlsbad, CA, USA). The extracted RNA was diluted 2-fold using DEPC-treated water. The concentration (μg/ml) and purity of RNA were determined using a U-2800 spectrophotometer (Hitachi High Technologies, Japan). Complementary DNA was synthesized from 100 ng of RNA by using SuperScript III First-Strand Synthesis SuperMix (Life Technologies, Carlsbad, CA, USA), according to the protocol. Then, a reaction mixture containing12.5 μl of SYBR select master mix for CFX (Applied Biosystems, Foster City, CA, USA), 1 μL (10 pmol) of forward and reverse primers of the target gene, 9.5 μL of DEPC-treated water, and 1 μl of cDNA was subjected to real-time PCR analysis (CFX96 Touch™ Real-Time PCR, Bio-Rad Laboratories Inc.). The primers used to detect the expression of target genes and their corresponding annealing temperatures are included in the Additional file 1.

### Histopathological analysis

Paraffin-embedded liver slices were sectioned (5 μm thick) and stained with hematoxylin and eosin dye. The histological changes were examined under a microscope (DIXI3000, Leica, Wetzlar, Germany). Based on the severity of lesions, scores were given for the major histopathological changes such as the degrees of hepatic necrosis, inflammation, balloon degeneration, and fatty degeneration according to Yang et al. [17]. Finally, the weighted scores were added and comparisons were made among groups.

### Statistical analysis

One-way analysis of variance followed by Duncan’s multiple range tests was conducted using SAS Software, Version 9.4 (SAS Inc., Cary, NC, USA). The results are expressed as the means ± standard error of at least three replicates. *P-*values less than 0.05 were considered statistically significant.

## Result

### LC/MS and GC/MS analysis

As shown in Fig. 2, the analysis of the non-fermented extract revealed various peaks with the highest at a retention time of 4.62 min (Fig. 2a and b, upper figures). However, it was disappeared after successive fermentation (Fig. 2a and b, lower figures). GC/MS analysis confirmed that the peak that disappeared upon successive fermentation represents 2-furan-carboxaldehyde.

### Effects of fermentation on amino acid content

The impact of fermentation on the amino acid content of HDE are summarized in Table 1. Primary fermentation contributed to the formation of amino acids such as citrulline, histidine, hydroxyproline, hydroxylysine, lysine, methionine, ornithine, and phenylalanine. However, arginine has emerged following secondary fermentation. Although successive fermentations resulted in alteration of the levels in almost all of the tested amino acids, significant changes were recorded in the GABA and glutamic acid content. The amount of GABA was markedly increased from 0.13 mg/ml to 92.24 mg/ml. In contrast, the level of glutamic acid was drastically reduced from 253.5 mg/ml to 2.8 mg/ml.

**Table 1:** Amino acid contents (mg/ml) of the HDE and FHDE

Amino acid (mg/ml)	Before fermentation	Primary fermentation	Secondary fermentation
1-Methylhistidine	ND^a^	ND	ND
3-Methylhistidine	ND	ND	ND
Alanine	0.259	2.835	0.442
Ammonia	0.270	1.982	1.396
Anserine	ND	ND	ND
Arginine	ND	ND	0.088
Aspartic acid	0.502	1.146	0.728
Carnosine	ND	ND	ND
Citrulline	ND	0.540	0.294
Cystathionine	ND	ND	ND
Cystine	0.125	0.216	0.137
Ethanol amine	ND	ND	ND
Glutamic acid	253.495	200.964	2.778
Glycine	0.069	0.113	0.181
Histidine	ND	0.045	0.073
Hydroxy proline	ND	0.128	0.043
Hydroxylysine	ND	0.041	0.058
Isoleucine	0.012	0.402	0.399
Leucine	0.017	0.283	1.023
Lysine	ND	1.123	0.811
Methionine	ND	0.214	0.215
Ornithine	ND	0.041	0.031
Phenylalanine	ND	0.404	0.280
Phospho ethanol amine	ND	ND	ND
Phosphoserine	ND	ND	ND
Proline	0.117	ND	1.001
Sarcosine	ND	ND	0.030
Serine	0.018	0.070	0.030
Taurine	ND	ND	ND
Threonine	0.006	0.094	0.025
Tyrosine	ND	0.237	0.155
Urea	ND	ND	ND
Valine	0.065	4.840	1.801
α-Amino adipic acid	0.053	ND	0.064
α-Amino-n-butyric acid	0.027	0.229	0.050
β-Alanine	0.005	0.075	0.507
β-Amino isobutyric acid	0.017	0.487	0.281
γ-Amino-n-butyric acid (GABA)	0.133	0.223	92.238

^a^*ND* is Not Detected

### Effects of treatment on feed intake and body weight

At the end of treatment, the body weight of mice in the normal control group (29.5 ± 0.6 g) was significantly (*P* < 0.05) higher than the other groups (Table 2). Likewise, treatment of mice with FHDE3 caused a significant (*P* < 0.05) body weight gain relative to the ethanol-treated mice. However, the differences in the feed intake and liver index (ratio of liver to body weight) were not significant among groups.

**Table 2:** Effects of FHDE on the mean of body weight, feed intake, and liver pathology score

	NRC	NC	FHDE1	FHDE3	HDE
Initial body weight (g)	21.5 ± 0.0	21.6 ± 0.4	20.4 ± 0.1	20.7 ± 0.2	21.4 ± 0.3
Final body weight (g)	29.5 ± 0.6^a^	25.6 ± 0.2^c^	26.4 ± 0.2^bc^	27.0 ± 0.3^b^	27.4 ± 0.2^b^
Weight gain (g)	8.0^a^	4.0^c^	5.9^bc^	6.3^b^	6.0^b^
Liver weight/Body weight	3.1%	3.3%	3.2%	3.1%	3.1%
Feed intake/each (g)	12.6	12.1	12.4	12.8	12.8

*NRC* normal control group, *NC* negative control group administered with the diet containing 3% ethanol, *HDE* administered with the diet containing 3% ethanol and 0.3% HDE, *FHDE1 and FHDE3* administered with the diet containing 0.1 and 0.3% FHDE, respectively in the diet containing 3% alcohol. Data are presented as mean ± SEM. Values with different letters indicate significant differences (*P < 0.05*)

### Plasma biochemical analysis

The impacts of FHDE on plasma biochemical parameters and lipid concentrations are presented in Table 3. A reduction (*P* < 0.05) in the activities of AST and ALT were detected following treatment with FHDE. Treatment reduced the activities of ALT and AST by 12 and 63%, respectively, compared to the levels in NC. Similarly, the plasma concentrations of LDL, TG, and FFA were significantly (*P* < 0.05) decreased in FHDE-treated mice. Interestingly, treatment with the fermented extract, especially with FHDE3, markedly reduced the plasma ALT and TG levels relative to the non-fermented extract that was used as a positive control.

**Table 3:** Effects of FHDE on plasma biochemical parameters and lipid concentrations

	NRC	NC	FHDE1	FHDE3	HDE
AST (U/ml)	52.35 ± 2.12^a^	68.70 ± 1.86^b^	61.26 ± 1.96^c^	59.88 ± 2.25^c^	61.92 ± 1.36^c^
ALT (U/ml)	17.42 ± 0.45^a^	32.92 ± 1.30^b^	16.90 ± 1.07^a^	12.24 ± 0.71^c^	17.09 ± 0.88^a^
Free fatty acid (%)	0.17 ± 0.01^a^	0.22 ± 0.01^b^	0.16 ± 0.01^a^	0.16 ± 0.01^a^	0.20 ± 0.01^abc^
LDL-Cholesterol (μg/μl)	1.68 ± 0.04^a^	2.13 ± 0.11^b^	1.61 ± 0.13^a^	1.65 ± 0.16^a^	1.74 ± 0.05^a^
HDL-Cholesterol (μg/μl)	3.09 ± 0.01^a^	3.01 ± 0.01^b^	3.03 ± 0.02^b^	3.06 ± 0.01^ab^	3.03 ± 0.01^b^
Triglyceride (g/mol)	1.60 ± 0.03^ad^	1.86 ± 0.02^bd^	1.66 ± 0.06^abd^	1.48 ± 0.06^ac^	1.76 ± 0.07^d^

*NRC* normal control group, *NC* negative control group administered with a diet containing 3% ethanol, *HDE* administered with the diet containing 3% ethanol and 0.3% HDE, *FHDE1 and FHDE3* administered with the diet containing 0.1 and 0.3% FHDE, respectively in the diet containing 3% alcohol. Data are presented as mean ± SEM of triplicate experiments. Values with different letters indicate significant differences (*P < 0.05*)

### Quantitative real-time PCR analysis (qRT-PCR)

**Fig. 3:**
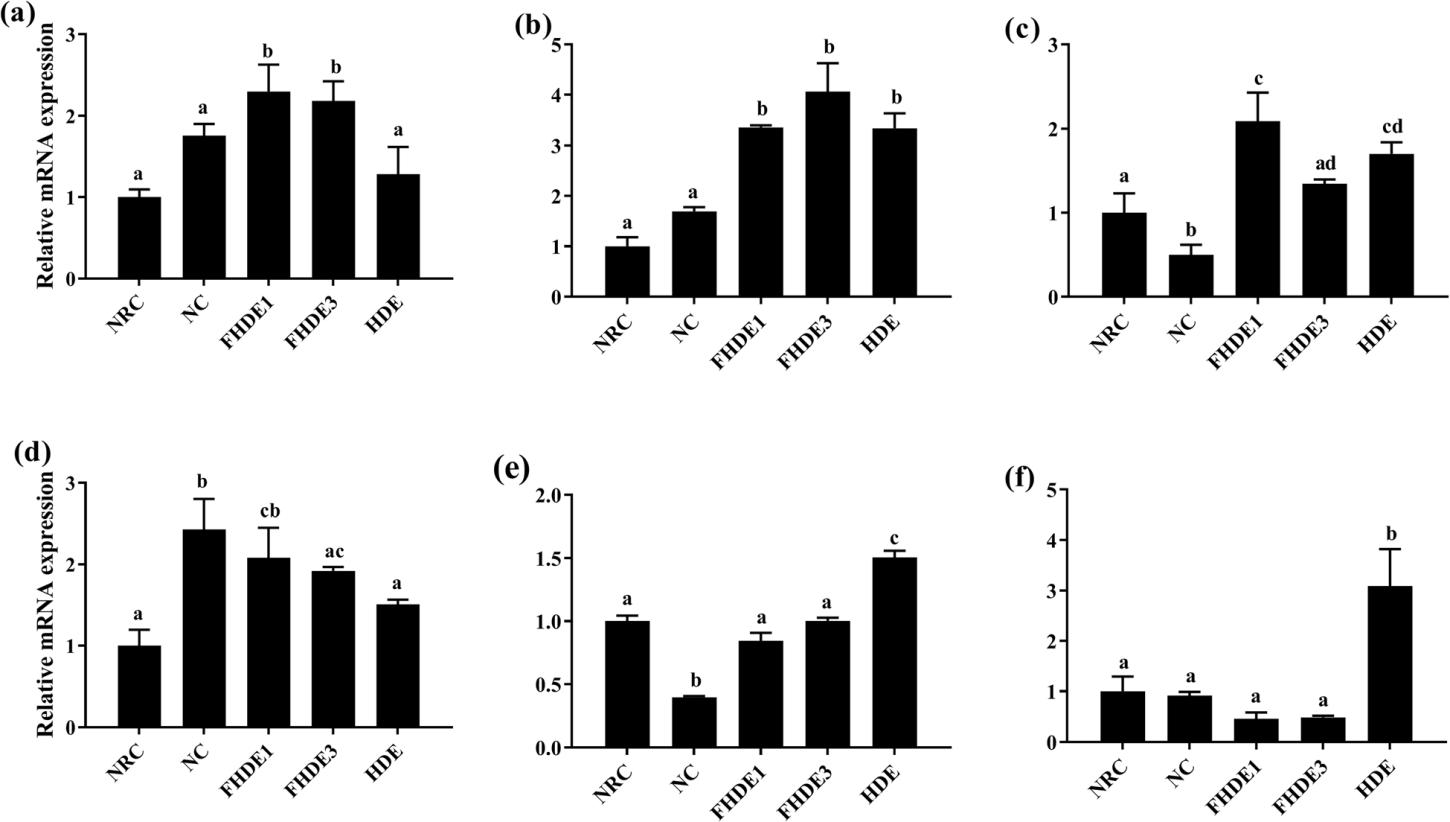
Effects of FHDE on the gene expression of alcohol dehydrogenase (*Adh1*), aldehyde dehydrogenase (*Aldh2*), catalase (*CAT*), cytochrome P4502E1 (*CYP*_*2*_*E*_*1*_), superoxide dismutase (*SOD*), and glutathione peroxidase (*GPx1*). NRC, normal control group; NC, negative control group administered with the diet containing 3% ethanol; HDE, administered with the diet mixture containing 3% ethanol and 0.3% HDE; FHDE1 and FHDE3, administered with the diet containing 0.1 and 0.3% FHDE, respectively in the diet containing 3% alcohol. Data are presented as mean ± SEM of triplicate experiments. Bars with different letters indicate significant differences (*P < 0.05*)

**Fig. 4:**
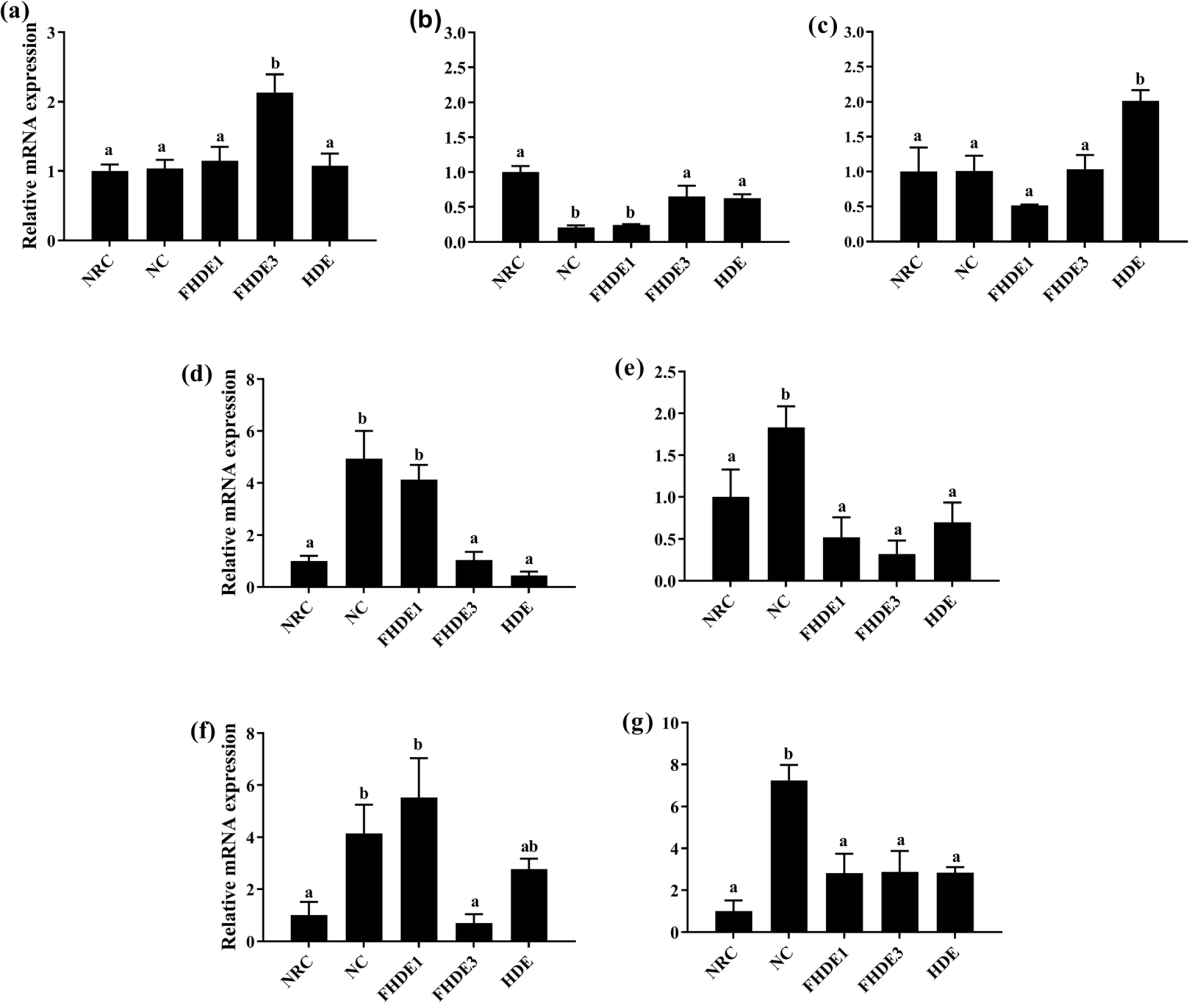
Gene expressions of peroxisome proliferator-activated receptor (*PPAR-α*), PPAR-γ coactivator (*PGC-1α*), carnitine palmitoyl-transferase (*CPT-1*), sterol regulatory element-binding protein (*SERBP-1c*), fatty acid synthase (*FAS*), stearoyl-CoA desaturase (*SCD1*), and acetyl-CoA carboxylase (*ACC*). NRC, normal control group; NC, negative control group administered with a diet containing 3% ethanol; HDE, administered with the diet mixture containing 3% ethanol and 0.3% HDE; FHDE1 and FHDE3, administered with the diet containing 0.1 and 0.3% FHDE, respectively in the diet containing 3% alcohol. Data are presented as mean ± SEM of triplicate experiments. Bars with different letters indicate significant differences (*P < 0.05*)

The expressions of genes that regulate the activities of enzymes related to alcohol dehydrogenation (*Adh* and *Aldh2*) and antioxidant enzyme activity (CAT and *SOD1*) were significantly upregulated (*P* < 0.05) in FHDE-treated groups with respect to those in the NC (Fig. 3). Treatment upregulated the gene expressions of *Aldh2*, *CAT*, and *SOD1* by at least 50%*.* However, FHDE3 and HDE downregulated the expression of *CYP*_*2*_*E*_*1*_ compared to ethanol. The expression of genes related to fatty acid oxidation such as *PPAR-α* and *PGC-1α* were significantly increased (*P* < 0.05) in mice treated with FHDE3 relative to those treated with ethanol only. In contrast, FHDE significantly (*P* < 0.05) prevented the ethanol-induced upregulation of genes involved in the regulation of lipogenesis including *SREBP-1c*, *FAS*, *SCD-1*, and *ACC* (Fig. 4).

### Histopathological analysis and liver pathology score

**Fig. 5:**
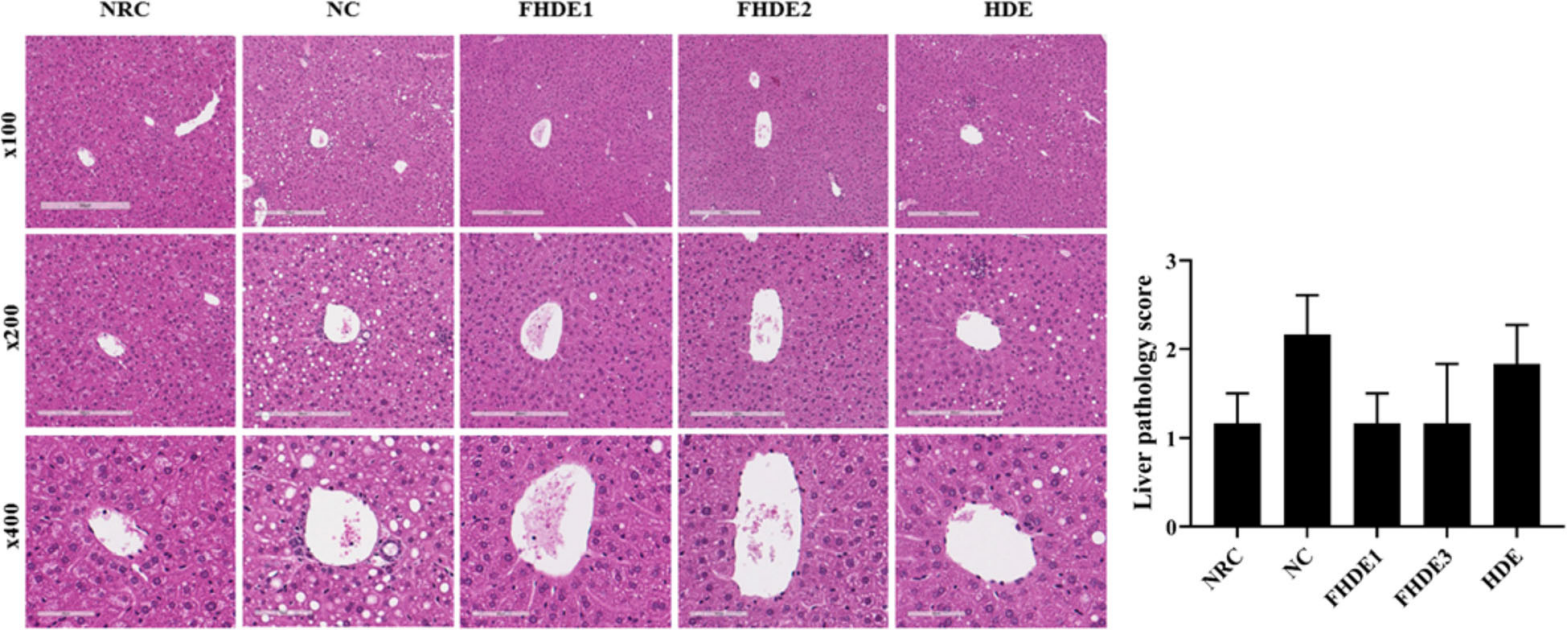
Effects of the EHDE on (**a**) histological changes and (**b**) pathology score of mice liver. NRC, normal control group; NC, negative control group administered with the diet containing 3% ethanol; HDE, administered with the diet mixture containing 3% ethanol and 0.3% HDE; FHDE1 and FHDE3, administered with the diet containing 0.1 and 0.3% FHDE, respectively in the diet containing 3% alcohol

The effects of treatment on histological changes of liver were evaluated and comparisons were made among groups (Fig. 5). The degree of hepatic lesions was evaluated and mean scores were given to the group, according to the severity of hepatic changes. Liver lobules in the normal control group (NRC) were scored as 1.2, as it showed slight steatosis. Marked infiltration of inflammatory cells, a slight degree of balloon degeneration, and fatty changes were evident in alcohol-treated mice with a mean liver score of 2.2. Except for a slight degree of inflammatory cell infiltration and occasional fatty changes, FHDE prevented most of the ethanol-induced hepatic lesions. The mean liver pathology scores of mice in FHED-treated groups were reduced by 53%, which was comparable to the normal control mice. Interestingly, FHDE was better in preventing the development of hepatic lesions compared to the most commonly used non-fermented product (HDE).

## Discussion

In the current study, we attempted to evaluate the hepatoprotective effects of FHDE against ethanol-induced liver injury and its underlying mechanisms in mice. Probiotic fermentation removed 2-furan-carboxaldehyde from HDE. This compound is reported to be carcinogenic and irritant with an LD_50_ of 65 mg/kg in rats [18]. In addition, fermentation caused an alteration in the levels of most of the tested amino acids with a marked increase and decrease in the amount of GABA and glutamic acid, respectively.

Although GABA is an inhibitory neurotransmitter in the central nervous system (CNS), the signaling mechanisms are also detected outside the CNS, such as endocrine cells of the pancreas [19], hepatocytes [20], and intestinal epithelial cells [21]. Norikura et al. [22] revealed the in vitro protective effects of GABA against ethanol-induced hepatotoxicity. A more recent study by Wang et al. [23] confirmed that pre-treatment of mice with GABA or muscimol (GABA receptor agonist) ameliorated the liver function and protected the liver from ethanol-induced toxicity through inhibition of the IRE1α- ASK1- JNKpro- apoptotic pathway. On the contrary, pretreatment with bicuculline (GABA receptor antagonist) caused deterioration of the liver function.

Chronic exposure of rats to alcohol is indicated to enhance the activity of glutamate dehydrogenase. Glutamate dehydrogenase dependent oxidation of glutamic acid results in an increase in reactive oxygen species (ROS) through the generation of excess superoxide anion and hydrogen peroxide from liver mitochondria. In addition to these characteristic features of alcohol intoxication, the damage will be further intensified as a result of the reduction in the level of mitochondrial catalase [24]. Therefore, fermentation-induced changes in the level of GABA and glutamic acid could contribute to the protective effects of FHDE from ethanol-induced hepatic damage.

Excessive alcohol intake has shown to induce dysbiosis and damage the intestinal mucosa. The damage might also be exacerbated by a further synthesis of alcohol and endotoxins by the intestinal bacteria. Finally, bacterial products translocate to the liver via the portal vein and induce liver damage [25–27]. GABA synergizes other mediators that regulate the intestinal immunity and provide a basis for clinical applications of nerve-induced immunity in the intestine [28, 29]. Previous studies have shown that intestinal inflammation or intestinal bacterial imbalance is closely related to alcohol-related liver injury [30, 31]. Therefore, the GABA enriched FHDE could reduce ethanol-induced liver injury by enhancing local immunity in the intestine.

FHDE treated mice gained more body weight compared to the ethanol-treated mice. The excess amount of monosaccharides present in the HDE might contribute to the significant body weight gain of FHDE-treated mice [3]. Besides, the activities of biomarkers of hepatic damage such as AST and ALT were markedly increased following ethanol intake but it was significantly reduced by both HDE and FHDE treatment. Chronic alcohol intake is known to cause alteration of hepatocyte function and necrosis of hepatocytes which results in elevated plasma AST and ALT levels [32]. Moreover, histopathological evaluation confirmed the preventive effects of FHDE from ethanol-induced inflammatory cell infiltration, fatty changes, and vacuolization suggesting its hepatoprotective effects.

Several enzymes such as alcohol dehydrogenase, aldehyde dehydrogenase, and Cytochrome peroxidase *CYP*_*2*_*E*_*1*_ contribute to the metabolism of alcohol and subsequent removal of its metabolic products from the body. FHDE treatment upregulated the expression of genes such as *Adh1* and *Aldh*, which are essential in the regulation of the activities of alcohol dehydrogenase and aldehyde dehydrogenase, respectively. Chronic alcohol intake suppresses the activity of alcohol dehydrogenase metabolic pathway and subsequently, *CYP*_*2*_*E*_*1*_ dominates the metabolic process. Unlike alcohol and aldehyde dehydrogenase, metabolism of alcohol via *CYP*_*2*_*E*_*1*_ produces metabolites that are toxic to hepatocytes such as superoxide anion and hydroxyl radicals [33, 34]. In this study, treatment with HDE and FHDE3 significantly reduced (*P < 0.05*) the expression of *CYP*_*2*_*E*_*1*_ gene. Therefore, FHDE could prevent ethanol-induced liver damage by enhancing the alcohol dehydrogenase metabolic pathway.

In alcoholic liver disease, excessive production of free radicals that emanates from the metabolism of alcohol and lipids result in oxidative stress [33]. The free radicals accumulate and interact with polyunsaturated fatty acids in the bio-membrane, which then results in lipid peroxidation and membrane damage. The problem is more pronounced in mice lacking antioxidant enzymes such as catalase, superoxide dismutase, and glutathione peroxidase [35]. In this study, the expression of *CAT* that regulates the activities of catalase was markedly up-regulated in the FHDE-treated mice compared to mice treated with ethanol, only. While the expressions of *SOD1* and *GPx1* genes that regulate the activities of superoxide dismutase and glutathione peroxidase, respectively were not affected by FHED-treatment. Interestingly, the expressions of *CAT*, *SOD1*, and *GPx1* were up-regulated in the HDE-treated mice with respect to the ethanol-treated mice. Therefore, the antioxidant potential of FHDE might also protect mice from ethanol-induced oxidative damage by enhancing the activities of antioxidant enzymes.

PCR assay demonstrated that the FHDE significantly (*P < 0.05*) downregulated the expressions of sterol regulatory element-binding protein (*SREBP-1C)*, fatty acid synthase (*FAS*), stearoyl-CoA desaturase 1 (*SCD*), and (acetyl-CoA carboxylase) *ACC* genes compared to the effects of ethanol. In contrast, the expressions of Peroxisome proliferator-activated receptor-γ (*PPAR-α)* and *PPAR*-*γ*-coactivator (*PGC-1α*) genes reduced significantly. Excessive accumulation of metabolic end-products of alcohol interferes with Adenosine monophosphate-activated protein kinase (AMPK), PPAR-α, and CPT-1 which are essential regulators of some of the enzymes involved in fatty acid oxidization in the liver [1, 34, 36]. A study by Chaung et al. [37] demonstrated that ethanol suppresses *PGC-1α* that causes liver injury through inhibition of the expressions of ROS-scavenging enzymes. Previous studies also confirmed that AMPK dependent down-regulation of *SREBP-1c* reduces the expression of *ACC* and FAS. Subsequently, lipogenesis and fatty acid oxidation become inhibited and enhanced, respectively [38, 39]. Besides, *SCD-1* is known to play a critical role in lipid-mediated signaling and the formation of hepatic steatosis [40]. Supporting the above findings, plasma biochemical analysis confirmed marked reduction of LDL-C, FFA, and TG levels, suggesting the protective effects of FHDE from the development of fatty liver mainly through its hypolipidemic effect. In agreement with this study, Kim et al. [6] reported the ameliorative effects of HDE on oleic acid-induced steatosis through activation of *AMPK* and *CPT-1* pathway. Therefore, FHED might prevent mice from the development of steatosis by up-regulating and down-regulating the expression of genes, which are essential in fatty acids oxidation and lipogenesis, respectively.

## Conclusions

*H. dulcis* is well known for its protective effect from alcohol-related liver injury. The current study demonstrated the presence of 2-furan-carboxaldehyde, a toxic metabolite, in the crude HDE. Successive fermentation with probiotics abolished the toxic metabolite and ameliorated the GABA content of the extract. Interestingly, FHDE protected the liver from ethanol-induced damage mainly by enhancing alcohol dehydrogenation, suppressing lipogenesis, and preventing oxidative damage in mice.

## Supplementary information

**Additional file 1.** List of primers used for assessing the expression of target genes that regulate the activities of enzymes related to alcohol dehydrogenation, antioxidant activity, fatty acid oxidation, and lipogenesis.

## Data Availability

All data generated or analyzed during this study are included in this published article.

## Abbreviations

ACC: Acetyl-CoA carboxylase
Adh1: Alcohol dehydrogenase
Aldh2: Aldehyde dehydrogenase
ALT: Alanine aminotransferase
AST: Aspartate aminotransferase
CAT: Catalase
CPT-1: Carnitine palmitoyltransferase
CYP_2_E_1_: Cytochrome P450_2_E_1_
FAS: Fatty acid synthase
FFA: Free fatty acid
FHDE: Fermented *H. dulcis*
GABA: Gamma-Amino-n-butyric acid
GPx1: Glutathione peroxidase
HDE: *H. dulcis* extract
LDL: Low-density lipoprotein
PGC-1α: Peroxisome proliferator-activated receptor-γ (PPAR-γ)-coactivator 1α
PPAR-α: Peroxisome proliferator-activated receptor-alpha
ROS: Reactive oxygen species
SCD-1: Stearoyl-CoA desaturase 1
SOD1: Superoxide dismutase
SREBP-1C: Sterol regulatory element-binding protein
TG: Triglycerides
