# Supplementary material for: Hepatoprotective effects of gamma-aminobutyric acid-enriched fermented Hovenia dulcis extract on ethanol-induced liver injury in mice
Source: BMC Complement Med Ther. 2020 Mar 6;20:75. doi: 10.1186/s12906-020-2866-0 (PMC7076742; doi:10.1186/s12906-020-2866-0)
Supplement: Supplementary file 1 — Additional file 1. List of primers used for assessing the expression of target genes that regulate the activities of enzymes related to alcohol dehydrogenation, antioxidant activity, fatty acid oxidation, and lipogenesis. [file 12906_2020_2866_MOESM1_ESM.docx]

Additional file 1. Primer sequence and melting temperature (°C) for the target genes

| Name | Forward | Reverse | Tm (°C) | Reference |
| --- | --- | --- | --- | --- |
| *PPAR-α* | CGAAGACAAAGAGGCAGAGG | TGATGTCACAGAACGGCTTC | 58 | [41] |
| *CPT-1* | ACTCCTGGAAGAAGAAGTTCA | AGTATCTTTGACAGCTGGGAC | 58 | [42] |
| *ACC* | ACGAGCACACACAGTCCATG | GATGACCTCTGGATGTTCTTG | 59 | [41] |
| *SREBP-1c* | GTAGCGTCTGCACGCCCTA | CTTGGTTGTTGATGAGCTGGAG | 57 | [43] |
| *SCD-1* | CCTACGACAAGAACATTCAATC | TTCTCTTAATCCTGGCTAAGAC | 59 | [41] |
| *FAS* | GGCTCAGCATGGTCGCTT | CTCCCGCCAGCTGTCATT | 57 | [43] |
| *Adh1* | GTTGGAGAAGGGGTGACTTG | AGATCGCTTCGGCTACAAAA | 58 | [44] |
| *Aldh2* | GCCTCAGGTGGATGAAACTC | CGGTGGGCTGGATAAGTAG | 58 | [44] |
| *CAT* | CGCCACATGAATGGATATGGA | GGTTTTTGATGCCCTGGTCA | 58 | [45] |
| *GPX1* | TTTCCCGTGCAATCAGTTCG | GGTGAGCCTTCTCACCATTC | 59 | [46] |
| *SOD1* | GGGTTCCACGTCCATCAGTA | ATTGCCCAGGTCTCCAACAT | 59 | [47] |
| *CYP2E1* | CGCATGGAACTGTTTCTGC | CAATTGTAACAGGGCTGAGGTC | 59 | [48] |
| *PGC-1α* | AGCCGTGACCACTGACAACGAG | GCTGCATGGTTCTGAGTGCTAAG | 58 | [41] |
| *β-actin* | TGACAGGATGCAGAAGGAGAT | CTCCTGCTTGCTGATCCACAT | 58 | [43] |
